# Anemia Mukt Bharat Index: Methodology and State Rankings of Iron and Folic Acid Supplementation Coverage in India, 2018–2019 to 2022–2023

**DOI:** 10.9745/GHSP-D-24-00077

**Published:** 2025-12-31

**Authors:** Zoya Ali Rizvi, Jitendra Singh, Preetu Mishra, Abhishek Kumar, Avi Saini, Narendra Patel, Neha Agarwal, Kapil Yadav, William Joe

**Affiliations:** a Ministry of Health and Family Welfare, Government of India, Delhi, India.; b Institute of Economic Growth, Delhi, India.; c UNICEF India Country Office, Delhi, India.; d FLAME University, Pune, India.; e All India Institute of Medical Sciences, Delhi, India.

## Abstract

In 2018, the Government of India launched the Anemia Mukt Bharat (AMB) program to accelerate reductions in the prevalence of anemia among children aged 6–59 months, children aged 5–9 years, adolescents aged 10–19 years, pregnant women, and lactating mothers through 6 programmatic interventions and 6 institutional mechanisms. We describe the process of computing the AMB index, aimed at providing timely and systematic information on iron and folic acid (IFA) supplementation coverage across these groups to aid in evaluating the effectiveness of the program. This study presents data from fiscal year 2018–2019 to 2022–2023 on IFA supplementation coverage among these 5 groups. We calculated the AMB index that provides an average for IFA supplementation coverage for target groups. Data on the target groups were acquired from the AMB dashboard, and information on IFA supplementation coverage was sourced from the health management information system. The AMB index confirmed that between 2018-2019 and 2022–2023 IFA supplementation coverage increased overall in India by 22.1 percentage points, from 35.5% to 57.6%. During this period, IFA supplementation coverage increased for all target groups including pregnant women, children aged 6–59 months, children 5–9 years, adolescents aged 10–19 years, and lactating mothers. The supply chain management and reporting of the data on the portal were among the key factors that substantially impacted the IFA supplementation coverage. The IFA supplementation coverage will significantly increase if the IFA supply chain and reporting standards improve. We discuss the policy implications and suggestions to improve the overall IFA supplementation coverage across India.

## Abstract (precis)

The Anemia Mukt Bharat (AMB) index provides valuable insights into the overall progress and challenges of the AMB program in India and a simple and comprehensive way to evaluate iron and folic acid supplementation coverage across groups.

## Teaser

**Key Findings:**

- The Anemia Mukt Bharat (AMB) program has contributed to an increase in iron and folic acid (IFA) supplementation coverage across India between fiscal year 2018–2019 and 2022–2023, indicating a positive impact on addressing anemia.
- Significant improvements in IFA supplementation coverage were observed among specific groups, such as children, lactating mothers, and pregnant women, emphasizing the need for targeted interventions.
- The study underscores the crucial role of monitoring tools such as AMB scorecards to regularly review and take course corrections at the national and state levels. It is essential that these tools are user-friendly, concise, and produced on a quarterly basis.

**Key Implications:**

- The AMB index provides a simple and comprehensive tool for program evaluation, allowing for informed decision-making and resource allocation at both national and state levels.
- Recognizing variations in IFA supplementation coverage among states, targeted interventions may be tailored to regional needs. Understanding these regional differences is crucial to optimize the effectiveness of initiatives aimed at reducing anemia.

## INTRODUCTION

Anemia, a condition where the number of red blood cells or their oxygen-carrying capacity is insufficient to meet physiological requirements,^1^ is a recognized global health problem and a major cause of morbidity and mortality among women and children in low- and middle-income countries.^2^ The World Health Organization’s global estimates report that 40% of children aged younger than 5 years, 37% of pregnant women, and 30% of women aged 15–49 years are anemic.^3^ The severity of anemia and the linked significant health, developmental, and economic impacts underscore the necessity for an intensified, multifaceted approach to address anemia.^4^^–^^8^ Iron and folic acid (IFA) supplementation is one of the primary interventions for preventing and managing iron deficiency anemia.

Studies from various low- and middle-income countries have shown that IFA supplementation can significantly improve hemoglobin levels and reduce the prevalence of anemia among pregnant women and children.^9^^–^^11^ The National Iron Plus Initiative and the Weekly Iron Folic Acid Supplementation program in India have contributed to increasing awareness about anemia and IFA supplementation.^12^^,^^13^ Suboptimal compliance to IFA supplementation has been a major challenge globally, with studies reporting compliance rates lower than 80% in many countries. In India, challenges include low compliance rates, particularly among pregnant women, misconceptions about IFA tablets, and side effects leading to discontinuation of the supplementation.^14^^,^^15^

IFA interventions have demonstrated both successes and challenges in tackling anemia globally and in India. While there have been notable successes with school-based programs and high compliance rates during pregnancy in some contexts, challenges persist.^12^ Inconsistent implementation due to factors like school absences and school holidays can hinder program effectiveness.^16^ A study by Berry et al.^17^ highlighted the challenges in implementing IFA supplementation programs in educational settings in India, emphasizing the need for strategies to improve adherence and address logistical barriers.

Poor coverage, particularly in India, has resulted in a stagnant prevalence of anemia despite ongoing efforts.^12^^,^^16^ Barriers to compliance, including lack of counseling, forgetfulness, and side effects (e.g., bloating), further contribute to the challenges.^16^^,^^18^ A study by Ahmed et al.^19^ emphasized the importance of formative research in designing effective IFA interventions for pregnant women, particularly in the first trimester of pregnancy. Supply chain issues, such as inadequate forecasting, inventory management, and fixed distribution schedules, also impede progress.^12^ In addition, low awareness of anemia, perceived low risk, negative social influences, and restrictive gender norms further exacerbate the challenges in promoting IFA interventions. Addressing these multifaceted challenges requires a comprehensive approach that tackles implementation inconsistencies, improves coverage, enhances compliance, strengthens supply chains, raises awareness, and addresses sociocultural barriers.^16^

According to the latest National Family Health Survey, prevalence of anemia in India is 67% among children 6–59 months old, 59% among adolescent girls 15–19 years old, 31% among adolescent boys, 57% among women of reproductive age (WRA), 52% among pregnant women, and 57% among lactating women.^20^ Compared to data from the previous National Family Health Survey in 2015–2016, the prevalence of anemia increased among most groups.^21^

### Anemia Mukt Bharat Program

In 2018, the Ministry of Health and Family Welfare (MOHFW) launched the Anemia Mukt Bharat (AMB) program as a component of the POSHAN Abhiyaan (National Nutrition Mission) to reduce the prevalence of anemia across different groups of individuals. AMB uses 6 key interventions to address anemia in 6 groups through 6 institutional mechanisms.

The 6 interventions comprise:

1. Providing prophylactic IFA supplementation to compensate for the iron deficiency in the body
2. Deworming with albendazole tablet for parasitic worm infections (e.g., hookworms, roundworms, and whipworms)
3. Conducting intensified year-round behavior change communication campaigns, including ensuring delayed cord clamping in newborns
4. Testing for anemia using digital methods and point-of-care treatment and diagnosing and treating anemia using advanced and digitized methods for early diagnostics, with a special focus on pregnant women and children
5. Mandating the availability of IFA fortified foods in government-funded public health programs
6. Addressing the nonnutritional causes of anemia in endemic pockets with special focus on malaria, hemoglobinopathies, and fluorosis

The 6 groups are:

1. Children aged 6–59 months
2. Children aged 5–9 years
3. Adolescents aged 10–19 years
4. WRA aged 20–49 years
5. Pregnant women
6. Lactating women

To intensify program implementation and accountability efforts, 6 institutional mechanisms^22^ are engaged:

1. Intraministerial coordination
2. Interministerial coordination
3. A national AMB unit
4. National centers of excellence
5. Strengthening of supply chain and logistics
6. Development of an AMB dashboard and digital portal

The AMB dashboard is a dedicated digital portal that provides the latest data for reporting, monitoring, and review, as well as information and resources related to anemia reduction for India.

The AMB program aimed to provide IFA supplementation to approximately 650 million individuals during the fiscal year (FY) 2023–2024. Table 1 summarizes the doses and IFA supplementation requirements for each of the 5 groups.^22^ Pregnant women, lactating mothers, and children are catered through different service delivery platforms like health centers, village health sanitation and nutrition days, and Anganwadi centers. Children aged 5–9 years and adolescents aged 10–19 years who attend school receive IFA supplementation through schools. Out-of-school adolescent girls receive supplementation through Anganwadi centers or accredited social health activists. All groups receive IFA supplementation-related services under the AMB program.

**TABLE 1.** AMB Program Guidelines for Prophylactic Doses and IFA Requirement for Different Groups, India

Groups	Prophylactic Dose	Regimen and Composition	Total Drug Requirement, Yearly
Children 6–59 months old	Biweekly, 1 ml of IFA syrup	1 mL IFA syrup contains 20 mg elemental iron + 100 mcg FA	2 syrup bottles
Children 5–9 years old	Weekly, 1 IFA tablet	1 pink^a^ sugar-coated tablet contains 45 mg elemental iron + 400 mcg FA	52 tablets
Adolescents 10–19 years old^b^	Weekly, 1 IFA tablet	1 blue^a^ sugar-coated tablet contains 60 mg elemental iron + 500 mcg FA	52 tablets
Pregnant women	Daily, 1 IFA tablet	1 red^a^ sugar-coated tablet contains 60 mg elemental iron + 500 mcg FA	180 tablets
Anemic pregnant women	Daily, 2 IFA tablets	1 red sugar-coated tablet contains 60 mg elemental iron + 500 mcg FA	360 tablets
Lactating mothers	Daily, 1 IFA tablet	1 red sugar-coated tablet contains 60 mg elemental iron + 500 mcg FA	180 tablets
Women of reproductive age 20–49 years old	Weekly, 1 IFA tablet	1 red sugar-coated tablet contains 60 mg elemental iron + 500 mcg FA	52 tablets

Abbreviations: AMB, Anemia Mukt Bharat; IFA, iron and folic acid; FA, folic acid.

^a^ The pink, blue, or red coloring of the tablets is recommended by the Ministry of Health and Family Welfare for easy identification of the tablets for each group.

^b^ Including both in-school boys and girls and out-of-school girls.

Source: AMB operational guidelines, 2018.^22^

The AMB program aimed to provide IFA supplementation to approximately 650 million individuals during the fiscal year 2023–2024.

Measuring IFA supplementation coverage was an impressive task and has been influenced by program design, approach toward community-based delivery, training of health care workers, counseling of individuals, and maintaining a continuous supply of IFA supplements. The key performance indicators that were available in the health management information system (HMIS) of the MOHFW provided information on the IFA supplementation coverage numbers for each group but not on the target number for each group of states/union territories (UTs). These indicators were also not consolidated for the program review. Therefore, for better comparison, it was critical to consolidate the data sets with denominators (the number of individuals targeted) for each group.

Accordingly, following the launch of the AMB program, a 1-page, user-friendly, and color-coded scorecard, including all the AMB indicators visible at a glance, was developed for program officials and rapid reviews. The AMB scorecard is used by national and state program officials to easily review the performance of the IFA supplementation coverage across states and districts and across 5 target groups. (WRA were not included due to the nonavailability of data.)

We aimed to (1) identify a suitable tool for measuring the AMB program’s overall performance in improving IFA supplementation coverage and in addressing anemia in the 5 groups; (2) examine the IFA supplementation coverage among the 5 groups and across Indian states/UTs, ranking their performance from FY 2018–2019 to FY 2022–2023; and (3) evaluate the program’s impact since its implementation. We also discuss the policy implications of these findings.

## METHODS

We illustrate the method of computing the IFA supplementation coverage among various groups; explain the method of computing the AMB index to provide timely and systematic information about IFA supplementation coverage across groups; and explain how the ranking of states/UTs is determined in the AMB scorecard and used to assess the progress and performance of the program.

### Computing Iron and Folic Acid Supplementation Coverage

IFA supplementation coverage is reported through the HMIS, which is an online portal of the MOHFW that provides information on all health indicators at block, district, state, and national levels. The data are uploaded directly at the health facility level in all states/UTs to support planning, management, and decision-making.

The AMB performance indicators reported in the HMIS, along with the numerators and denominators used for each group, are provided in Table 2. The numerator value, which was acquired from the HMIS, is the total number of individuals in each group provided with IFA tablets up to the reported month as per the categorization in the AMB guidelines. The denominator value, which was acquired from the AMB dashboard, is the total number of target individuals of the specific group and period. The IFA supplementation coverage was computed in percentage by dividing the numerator and denominator of each specific group individually. A performance scorecard ranking was created to assist each state/UT in evaluating how well each district reported and uploaded data to the HMIS portal. To eliminate the issue of overreporting, a ceiling of 95% was used for IFA supplementation coverage values. Missing values indicated the unavailability of data. This was a common occurrence and could have had a significant effect on the conclusions drawn from the data. To avoid inference with data, missing values were treated as zero values when calculating the index value, which reduced error and provided a clearer depiction of the index’s value.

**TABLE 2.** AMB Program Performance Indicators^a^ Reported in the HMIS, India

**Group** ^b^	**HMIS Indicators**	**Provided IFA (Numerator)**	**AMB Target (Denominator)**
Children aged 6–59 months	9.9: Number of children aged 6–59 months provided 8–10 doses (1 mL) of IFA syrup (biweekly)	Number of children aged 6–59 months provided 8–10 doses (1 mL) of IFA syrup (biweekly)	Total number of children aged 6–59 months (NIPI target)
Children aged 5–9 years	23.1: Number of children covered under WIFS junior (5–9 years) provided 4–5 IFA tablets in schools23.3: Number of out-of-school children (5–9 years) given 4–5 IFA tablets at Anganwadi centers	Number of children covered under WIFS junior aged 5–9 years in school + number of out-of-school children (aged 5–9 years) provided 4–5 IFA tablets per month	Total number of children aged 5–9 years (NIPI target)
Adolescents aged 10–19 years	22.1.1.b: Number of boys (6th–12th class) provided 4 IFA tablets in schools 22.1.1.a: Number of girls (6th–12th class) provided 4 IFA tablets in schools 22.1.3: Number of out-of-school adolescent girls provided 4 IFA tablets at Anganwadi centers	Number of boys + girls in school + out-of-school girls aged 10–years provided 4 IFA tablets per month	Total 10–19 years in-school girls + 10–19 years in-school boys + 10-19 years out-of-school adolescent girls (WIFS target)
Pregnant women	1.2.4: Number of pregnant women provided full course 180 IFA tablets 1.1: Total number of pregnant women registered for ANC	Number of pregnant women provided full course 180 IFA tablets	Total number of pregnant women registered for ANC
Lactating mothers	6.3: Number of mothers provided full course of 180 IFA tablets after delivery	Number of mothers provided full course of 180 IFA tablets after delivery	Lactating mothers (live births–midyear population × crude birth rate)

Abbreviations: AMB, Anemia Mukt Bharat; ANC, antenatal care; HMIS, health management information system; IFA, iron and folic acid; FA, folic acid; NIPI, National Iron Plus Initiative; WIFS, Weekly Iron and Folic Acid Supplementation program.

^a^ Indicators are per the HMIS before March 2023; some are updated in new HMIS from April 2023.

^b^ Indicator for women of reproductive age under development.

### Process of Uploading Data on the Health Management Information System Portal

**FIGURE 1:**
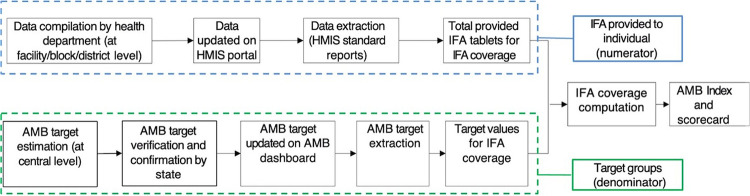
Processes Involved in Computing IFA Coverage and AMB Index, India Abbreviations: AMB, Anemia Mukt Bharat; HMIS, health management information system; IFA, iron and folic acid.

The HMIS data are reported at the state, district, and block levels. The indicators for children aged 6–59 months, pregnant women, and lactating mothers are reported at the facility level through available reporting formats (health subcenters) and uploaded at block-level health facilities (primary health centers or community health centers) by the data manager/medical officer. The indicators for children aged 5–9 years and adolescents aged 10–19 years are compiled at the district level by the education department and uploaded by district-level health facilities in the portal as collected from all blocks. The data for out-of-school children are reported by community childcare centers (Anganwadi centers), compiled by the block-level child development project officer, and shared with the block-level health functionary. The reporting mechanism might vary across states due to variations in the reporting systems of their health, integrated child development services, and education departments. The data collection process and subsequent updates on the HMIS portal are presented in Table 3 as per the AMB training toolkit,^23^ and processes for computing IFA supplementation coverage and AMB index are illustrated in Figure 1.

**TABLE 3.** Process of Data Flow Into the HMIS Portal for All Groups, India

**Group**	**Data Collection, Level and Person Responsible**	**Data Compilation Performed, Level and Person Responsible**	**Data Updated on HMIS Portal, Level and Person Responsible**
Children aged 6–59 months	Subcenter, ANM	PHC/CHC level, medical officer	At block level by BPO
Children aged 5–10 years and adolescents aged 10–19 years in school	School, schoolteachers	Education department at district level	At district level by DPO (health)
Children aged 5–10 years and adolescents aged 10–19 years out of school	Anganwadi center, AWW	Integrated child development services department, WCD	At block level by BMO/BPO
Pregnant women	Subcenter, ANM	PHC/CHC level, medical officer	At block level by BPO
Lactating mothers	Subcenter, ANM	PHC/CHC level, medical officer	At block level by BPO

Abbreviations: AMB, Anemia Mukt Bharat; ANM, auxiliary nurse-midwife; AWW, Anganwadi worker; BPO, block program officer; BMO, block medical officer; CHC, community health center; CDO, child development officer; DPO, district program officer; HMIS, health management information system; PHC, primary health center; WCD, women and child development.

Source: AMB training toolkit, 2019.

### Methods of Computing the Anemia Mukt Bharat Index

The AMB index is the average of the 5 key performance indicators for the different groups and serves as the basis for generating AMB scorecards. This index value is used to rank both states and districts based on their performance for each group. The AMB index is primarily based on the HMIS of the MOHFW and is reflected in the AMB dashboard. To compute the AMB index value, we used the composite index method, geometric mean method, and simple average method. The composite index aims to establish a metric that combines all the indicated factors or indicators and reflects the overall status/progress or distance from a set of quantifiable goals. For example, while computing the index, normalization of coverage is performed.^24^ In addition, the index is calculated as the arithmetic mean of the group indices for the state/district. The resulting index value falls between 0 and 1 and produces a negative coverage index for nonreported (blank) states or districts. The geometric mean is another way to find the average that is typically used for calculating growth rates, such as interest rates or population growth. The definition of the geometric mean is “the nth root of the product of n numbers.” The geometric index is computed by taking all groups’ nth root coverage values.

The simple average method gives equal weight to all groups. The ranking computed with the geometric mean method is significantly different from the simple average method. Also, some states have a zero (0) index value when computing it using the geometric mean method, which indicates zero IFA supplementation coverage. However, the ranking computed with the composite index method is somewhat similar to the simple average method. Table 4 and the Supplement provide a detailed comparison of the methods.

**TABLE 4.** Benefits and Limitations of Three Methods Used to Compute the Anemia Mukt Bharat Index

**Method**	**Benefits**	**Limitations**
Composite index	Normalizes coverage. Reflects overall status/progress. Allows for comparisons across different indicators.	Can produce a negative coverage index for nonreported states/districts. Complex to interpret. Can be sensitive to outliers.
Geometric mean	Useful for calculating growth rates. Less sensitive to outliers compared to the simple average method.	Can produce zero index value with zero coverage states. May not be suitable for data with zero values.
Simple average	Gives equal weightage to all groups. Simple to understand and interpret. Less sensitive to fluctuations in individual indicators.	May not be suitable for complex analyses. Requires different weights for groups. Can be influenced by outliers.

We chose the simple average method for its simplicity, interpretability, and alignment with the AMB program’s objectives that gave equal importance to all groups. This method allowed for a straightforward understanding of the overall IFA supplementation coverage and facilitated clear comparisons between states/UTs. While other methods may have been useful for specific types of analyses, the simple average method was deemed most appropriate for the purpose of this study.

To maintain the integrity of the AMB index and prevent improbable values, any coverage value exceeding 95% (including more than 100%) was recorded as 95%. This ceiling was used because, in real-life scenarios, 100% coverage is difficult to achieve, and it helped to reduce the effect of overreporting and ensured that the highest coverage reflected in the AMB index was 95%. All 28 states and 8 UTs in India were ranked based on the AMB index, whereby the state/UT with the highest index value was ranked first. The ranking was calculated separately for states and UTs because UTs have different health system structures and different budget allocation ratios compared to states.

The AMB index can be used to identify states/UTs that are performing well and those that need improvement in terms of IFA supplementation coverage. A higher AMB index value indicates better IFA supplementation coverage across the various groups. Program managers can use the AMB index and the state rankings to identify areas that need attention and to prioritize interventions. For example, if a state has a low AMB index ranking, it may indicate that there are gaps in the IFA supply chain or challenges in reaching certain groups. The AMB index can also be used to track progress over time and evaluate the effectiveness of interventions. By monitoring the AMB index, program managers can identify whether interventions are having the desired impact on IFA supplementation coverage.

### Quality Checks

The HMIS uses a multipronged approach to ensure data quality and minimize reporting errors. State and district officials conduct periodic site visits to verify data reporting practices directly at health care facilities. Scheduled supportive supervision visits and HMIS data audits are implemented to meticulously cross-check data and identify discrepancies. Furthermore, a comprehensive HMIS data validation tool is readily available to all states and districts. This tool facilitates data validation by pinpointing outliers, zero-reporting facilities, and other inconsistencies that may compromise data integrity.

It is acknowledged that the possibility of erroneous data entry cannot be entirely eliminated. However, the HMIS data validation tool serves as a critical safeguard to rectify and resolve these reporting errors. The calculation of the AMB index, as previously discussed, also incorporates measures to detect overreporting. The quality of data reported also depends on the number of facilities reporting each month, known as reporting efficiency. The AMB index also reflects the same for each district and helps program officers improve the reporting quality and, hence, the data quality in their respective districts.

## RESULTS

From FY 2018–2019 to 2022–2023, the AMB program increased IFA supplementation coverage across all groups.

### Number of Individuals Covered

**FIGURE 2:**
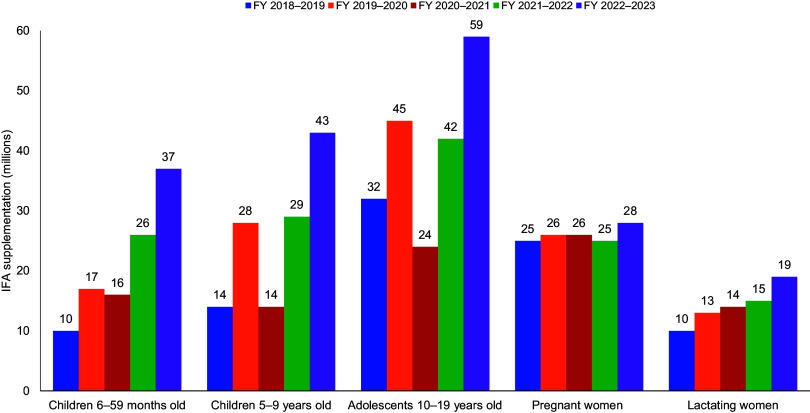
Total Number of Individuals Covered Under the AMB Program, India, by Fiscal Year Source: HMIS and AMB dashboard. Abbreviations: AMB, Anemia Mukt Bharat; FY, fiscal year; HMIS, health management information system; IFA, iron and folic acid.

The number of individuals covered under the AMB program substantially increased across all groups, with the most significant increase observed from 32 to 59 million for adolescents aged 10–19 years (Figure 2).

The number of individuals covered under the AMB program substantially increased across all target groups.

### Iron and Folic Acid Supplementation Coverage by Fiscal Year

**FIGURE 3:**
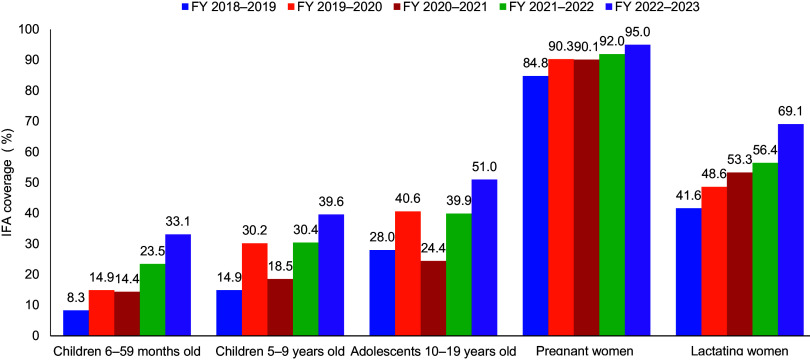
IFA Supplementation Coverage Under the AMB Program, India, by Fiscal Year Source: HMIS standard report. Abbreviations: AMB, Anemia Mukt Bharat; FY, fiscal year; HMIS, health management information system; IFA, iron and folic acid.

Figure 3 illustrates the increase in IFA supplementation coverage from FY 2018–2019 to 2022–2023 for all groups. The IFA supplementation coverage for FY 2022–2023 was highest for pregnant women (95.0%) and lowest for children aged 6–59 months (33.1%). In addition, the IFA supplementation coverage among lactating mothers consistently increased, indicating better coverage captured for the groups.

The t-test was conducted to show the quarterly (3-month) improvement in IFA supplementation coverage from FY 2018–2019 to 2022–2023 (Supplement Table S2). The t-test results showed statistically significant improvements (*P*<.05) across all target groups. The mean quarterly coverage for children aged 6–59 months increased by 16.15 percentage points (*P*=.00087). Similarly, significant improvements were observed for children aged 5–9 years (*P*=.00056) and adolescents 10–19 years (*P*=.00329). The *P* value represents the probability of observing the data if there were no real differences in coverage between the 2 periods.

### Iron and Folic Acid Supplementation Coverage by State and Union Territory

There was significant interstate variation in IFA supplementation coverage, particularly among all children (Table 5). There was a lower coefficient of variation for pregnant women (0.15) compared to other groups (children aged 6–59 months: 1.0; children aged 5–9 years: 0.76; adolescents aged 10–19 years: 0.59; and lactating mothers: 0.31).

**TABLE 5.** IFA Coverage and AMB Index, by State and Union Territory, India, FY 2022–2023

	**IFA Coverage, %** ^a^					
	**Children Aged 6–59 Months**	**Children Aged 5–9 Years**	**Adolescents Aged 10–19 Years**	**Pregnant Women**	**Lactating Mothers**	**AMB Index,%** ^b^
**States**						
Telangana	95.0	83.1	87.7	95.0	95.0	91.2
Tamil Nadu	72.1	95.0	95.0	95.0	92.3	89.9
Chhattisgarh	78.5	83.6	86.4	95.0	74.4	83.6
Andhra Pradesh	82.4	68.5	79.3	95.0	73.0	79.6
Madhya Pradesh	60.6	84.8	78.7	95.0	60.1	75.8
Odisha	57.2	55.0	73.6	94.8	65.7	69.3
Haryana	81.7	35.0	87.3	87.1	53.3	68.9
Maharashtra	59.8	41.2	40.1	95.0	76.2	62.5
Assam	31.0	49.2	61.8	95.0	75.2	62.4
Jharkhand	33.3	35.4	73.1	91.3	75.3	61.7
Gujarat	29.3	54.1	57.1	95.0	71.5	61.4
Rajasthan	61.9	19.3	42.2	95.0	71.9	58.1
Goa	7.5	23.3	78.9	83.5	83.2	55.3
Himachal Pradesh	34.4	47.6	45.5	80.9	62.8	54.2
Tripura	8.9	51.7	82.2	87.0	39.8	53.9
West Bengal	50.6	12.2	41.3	91.4	71.8	53.5
Karnataka	7.9	35.2	37.6	95.0	80.3	51.2
Punjab	18.1	34.2	71.8	72.7	55.9	50.5
Uttarakhand	9.4	43.4	38.0	89.6	71.3	50.3
Uttar Pradesh	1.1	51.9	51.9	95.0	44.0	48.8
Sikkim	5.8	5.3	20.2	87.4	95.0	42.7
Mizoram	0.4	29.8	70.5	73.3	31.1	41.0
Kerala	15.6	2.8	3.0	95.0	59.2	35.1
Bihar	3.4	7.0	18.8	81.1	44.6	31.0
Meghalaya	2.9	0.4	9.8	67.9	47.3	25.7
Arunachal Pradesh	0.2	0.5	0.6	74.1	51.6	25.4
Nagaland	0.0	0.6	7.3	60.7	36.4	21.0
Manipur	0.1	5.5	3.4	33.2	17.0	11.8
**Union Territories**						
Jammu and Kashmir	22.9	82.9	45.4	95.0	66.5	62.5
Puducherry	6.6	49.2	65.5	95.0	87.0	60.7
Dadra and Nagar Haveli and Daman and Diu	50.1	59.1	53.7	91.3	47.6	60.4
Chandigarh	1.2	18.0	88.9	95.0	95.0	59.6
Andaman and Nicobar Islands	43.9	57.9	42.7	63.6	61.8	54.0
NCT of Delhi	9.4	9.8	31.2	89.1	43.8	36.7
Ladakh	5.9	1.8	9.0	95.0	46.1	31.6
Lakshadweep	0.0	0.0	0.0	94.2	34.5	25.7
**All India**	**33.1**	**39.6**	**51.0**	**95.0**	**69.1**	**57.6**
Coefficient of variation	1.00	0.76	0.59	0.15	0.31	0.40

Abbreviations: AMB, Anemia Mukt Bharat; HMIS, health management information system; IFA, iron and folic acid.

^a^ Indicators are per HMIS before March 2023; some are updated in the new HMIS from April 2023. IFA coverage calculated from key performance indicator numerators and denominators in Table 2.

^b^ Simple mean of coverage values for 5 groups.

Source: Authors’ computation based on HMIS and AMB dashboard as on June 15, 2023.

**FIGURE 4:**
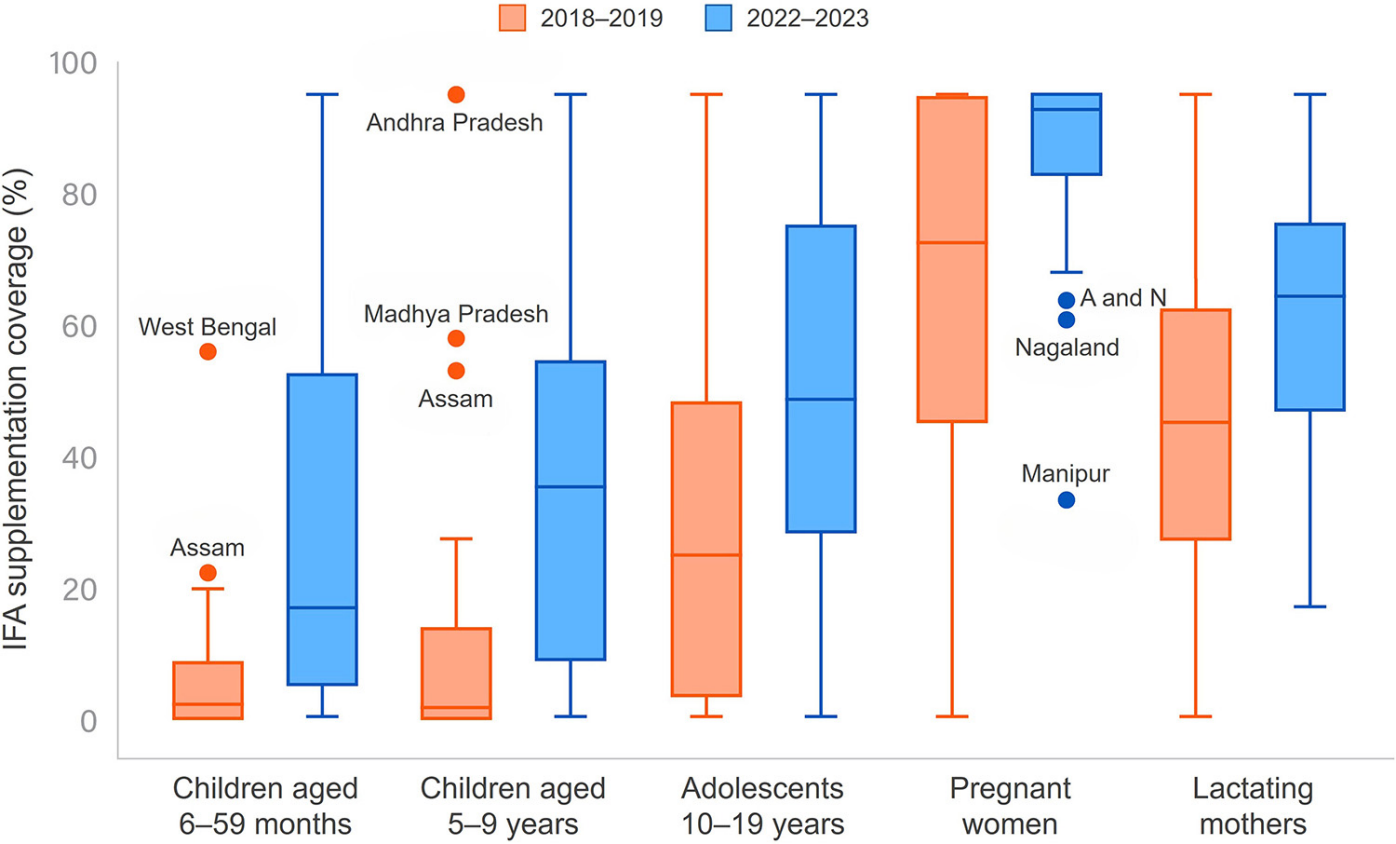
Boxplot of IFA Supplementation Coverage, India Abbreviations: A and N, Andaman and Nicobar; IFA, iron and folic acid.

Figure 4 highlights the increase in median IFA supplementation coverage for all groups, indicating an overall improvement in IFA supplementation coverage from FY 2018–2019 to 2022–2023. The highest median change was 33.3% among children aged 5–9 years, followed by 21.3% among adolescents aged 10–19 years. Assam and West Bengal for children aged 6–59 months and Andhra Pradesh, Madhya Pradesh, and Assam for children aged 5–9 years were notable outliers for high IFA supplementation coverage. Tamil Nadu, Telangana, and Haryana reported better performance and were the top 3 states for adolescents aged 10–19 years in FY 2022–2023 (Table 5). This age group among all children shows a median value of around 50% (i.e., half of the states are recording an IFA supplementation coverage of more than 50%).

### Anemia Mukt Bharat Index

The AMB index was computed as a simple average of IFA supplementation coverage for the different groups for FY 2022–2023 (Table 5). The AMB index value overall in India was 57.6% in 2022-23 for the country and shows an increase from 35.5% in 2018-19 (data for 2018-19 not displayed).

**FIGURE 5:**
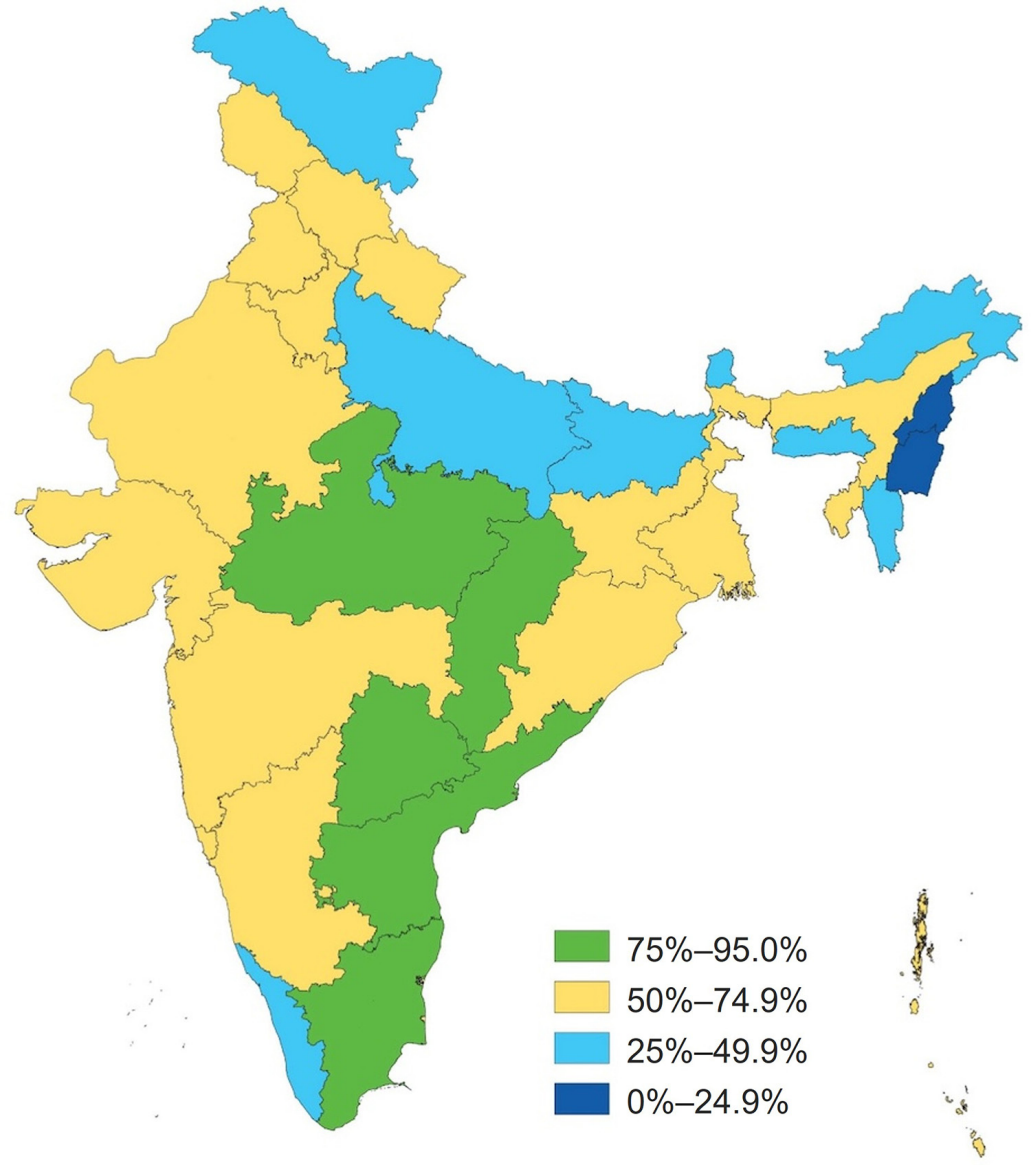
AMB Index Value Distribution, by State, India, Fiscal Year 2022–2023^a^ Abbreviation: AMB, Anemia Mukt Bharat. ^a^ Up to March 2023.

Figure 5 presents a state-level distribution of the AMB index for FY 2022–2023. The index values and rankings highlight the interstate variations in IFA supplementation coverage. Among states, Telangana ranked first with an index value of 91.2, and Manipur ranked last with an index value of 11.8. In UTs, Jammu and Kashmir ranked first (index value of 62.5), and Lakshadweep ranked last (index value of 25.7).

## DISCUSSION

Since the 1970s, the Government of India has been implementing various health programs to reduce the prevalence of anemia, including the AMB program since 2018. Reviews of any program to assess the performance and understand gaps are necessary to ensure desired goals are met. To review IFA supplementation coverage across target groups and to facilitate a simple yet comprehensive interpretation of progress, the AMB index was developed based on the HMIS indicators. States are ranked in descending order of mean coverage, with the state/UT reporting the best coverage ranked first.

The AMB scorecard summarizes 5 key performance indicators for target groups. The AMB scorecard is intended to help national and state program managers analyze program performance quarterly and plan corrective strategies accordingly. The key findings from the AMB scorecard exercise include the importance of comprehensive data for tracking program progress across groups. It has become a commonly used tool in program planning and reviews at all levels. As a result, it captures details on the supply and demand side of the IFA supplementation from the state level to the last-mile delivery points. Many states use this to finalize their budgets and procurement plans for IFA supplements to strengthen the availability and distribution of IFA tablets/syrup. States/UTs have since embraced the AMB scorecards, using them to highlight achievements. Nationally, scorecards were the preferred reference for anemia-related information in parliament, state trainings, and program reviews. The recommendations for improvement of the system are to include HMIS data for WRA, the sixth target group under the AMB program, and expand the scorecard and AMB index visualization tool to facility-level data.

The AMB scorecard is intended to help national and state program managers to analyze program performance quarterly.

In our analysis, IFA supplementation coverage increased across all groups after the implementation of the AMB program strategy from FY 2018–2019 to FY 2022–2023. It is important to consider the potential impact of the COVID-19 pandemic on the observed decline in IFA supplementation coverage during FY 2020–2021 for some groups. The pandemic disrupted health services and necessitated school closures and lockdowns that may have affected IFA tablet distribution and consumption. Studies have shown that the pandemic had a significant impact on the delivery of maternal and child health services, including IFA supplementation. The median IFA supplementation coverage increase observed could be attributed to several factors, such as focused interventions, better supply chain management, or efficient data reporting in these states. However, it is also essential to rule out the possibility of overreporting by implementing data quality checks.

While IFA supplementation coverage steadily improved for all target groups, there are still opportunities to further enhance program reach and effectiveness. The fundamental problem of suboptimal coverage has been partly associated with distribution gaps in the IFA supplement supply chain across various groups. The program has developed specific key performance indicators to review the status and address gaps in procurement and distribution through improved financing and coordination between state/UT governments.^25^^,^^26^ Figure 5 also emphasizes the need for targeted interventions and resource allocation to address the state-level variations and specific needs.

The AMB index revealed significant variation across states that could be attributed to a multitude of factors. Primarily, differences in program implementation at the state level play a significant role. This includes variations in the efficiency of state health systems, the reach of community-based delivery,^27^ the quality of health care worker training, and the consistency of IFA supplement supply.^12^ Additionally, the accuracy and timeliness of data reporting, efficient supply chain management, and effective coordination with other departments (e.g., education and women and child development) all contribute to higher AMB index scores.

Furthermore, states that prioritized reaching all groups, including children, adolescents, pregnant women, and lactating mothers, demonstrated better IFA supplementation coverage and higher scores. Investing in regular training and capacity-building for officials, actively involving the community in promoting IFA supplementation, and ensuring adherence to dosing protocols are also crucial for improving program effectiveness and achieving higher AMB index scores. However, there are still opportunities to further enhance program reach and effectiveness, particularly in addressing the suboptimal coverage associated with gaps in the IFA supply chain.

The Health Index^28^ by NITI Aayog serves as a valuable tool for tracking India’s progress in improving overall health. The AMB index specifically tracks the coverage of IFA supplementation among various groups, serving as a direct measure of the AMB program’s effectiveness in combating anemia, while the Health Index takes a broader approach, encompassing various health indicators to assess the overall health performance of Indian states and UTs. Comparing the 2 indices can help identify states that need targeted interventions to improve both IFA supplementation coverage and overall health performance.

The findings of Balarajan et al.,^29^ Wangaskar et al.,^30^ Pena-Rosas et al.,^31^ and Varghese et al.^32^ support the conclusion that anemia is a significant public health problem in India, requiring a comprehensive approach to address it effectively. The AMB program’s focus on IFA supplementation aligns with the recommendations of these studies for improving nutrition to combat anemia. However, our study highlights the challenges in achieving high IFA supplementation coverage and emphasizes the need for targeted interventions and improved program implementation to address anemia in India.

The utilities of the AMB index can be summarized as follows. Comprehensive use of AMB scorecards and index-based ranking is expected to boost implementation efforts and yield positive results in decreasing anemia prevalence. It supports the assessment of progress and performance and creates a robust interstate competition for improved performance. The AMB index assists in improving HMIS and AMB data reporting and timeliness, which is useful for states to engage with districts and improve compliance in reporting quality, coverage, and timeliness. Further, the AMB indicators under the HMIS system can establish a reliable mechanism to measure the program’s progress. Thanks to the AMB index, there has been a substantial focus on improving the count of individuals across various target groups (denominators for index computation). Major improvements have been noted in estimating coverage requirements for adolescents and school-going children. Strengthening IFA supplementation coverage is one of the core strategies, and, in this regard, the AMB index has drawn attention to both demand-side and supply-side concerns, underlying poor performance. The AMB index highlights the need for strategic budgeting and resource allocation decisions to enhance coverage, leveraging insights from the analysis of the previous year’s coverage data.

The association between IFA supplementation coverage and anemia prevalence should be tracked with respect to district and subdistrict levels. The AMB index is currently available for ranking district-level performance. However, in the future, leveraging regular subdistrict-level rankings will offer a more detailed perspective on the program’s implementation and impact. District program managers can pinpoint subdistricts with low IFA supplementation coverage, enabling targeted corrective efforts. Additionally, this approach will enhance the understanding of contextual factors influencing program implementation and performance at the subdistrict level. The subdistrict level AMB index could also serve as a useful measure to identify gaps in supply or demand and provide guidance on where to focus to improve the overall coverage from the micro-level. In this regard, additional training of data managers and program officers to enable them to deliver prevention and control programs with more precise data and analysis over time will be necessary. Training content on data management can improve reporting and data analysis, providing a more complete picture of the program at the state/UT level and resulting in more significant change and development of actionable points.

### Study Limitations

While the analysis of the AMB index offers a comprehensive overview, it is subject to certain limitations that warrant consideration. First, the use of HMIS data for calculating coverage projections, collected at the state level, may have introduced inaccuracies due to reporting delays, nonreporting districts, and missing data. Moreover, data overlaps at the district level could have led to duplications. Data entry errors and reporting inconsistencies at the facility level may have also existed and may have affected the accuracy of the AMB index. For example, inaccurate recording of the number of individuals receiving IFA supplements can lead to an overestimation or underestimation of the actual coverage. Similarly, inconsistencies in data reporting across different districts or states can affect the comparability of the AMB index and the resulting state rankings. While the 95% ceiling helps mitigate the impact of overreporting, it does not completely eliminate the potential for errors. Therefore, it is crucial to acknowledge that the AMB index provides an approximation of the actual IFA supplementation coverage, and the interpretation of the index should consider the potential for data-related limitations. Second, the reporting efficiency of the districts (number of months reported in a year) may cause a difference between actual coverage and reported coverage. However, the information on facilities that reported for less than 12 months is available and shared with authorities for necessary action. Third, the HMIS provides data on IFA supplementation for only 5 of 6 groups (WRA not included), excluding a large number (264.7 million) of AMB individuals from the IFA supplementation coverage analysis.

Furthermore, the HMIS data captures aggregated data on IFA supplementation, limiting the scope of analysis. It does not include information on the specific program service delivery platforms used by states to reach the target groups, such as the various delivery mechanisms and their effectiveness. This limits the ability to examine the impact of different program service delivery platforms on IFA supplementation coverage. Additionally, the study acknowledges that the AMB index is based on the HMIS data, and the information on the gap between receiving and consuming IFA tablets is neither exhaustive nor explicit. Many indicators related to perceptions about IFA supplementation among women could not be captured in this study. More primary research understanding the roles of community health workers in social mobilization and motivating women to reach a higher IFA consumption level is required.

Iron deficiency is certainly one of the major contributors to anemia, but many other factors, like blood dyscrasias and deficiencies in folate, Vitamin B12, and Vitamin A, can also play a role in the development of this condition. These vitamins are crucial for making hemoglobin and red blood cells, and without them, the whole process gets disrupted. Chronic inflammatory conditions can also play a role, leading to a specific type of anemia. Lastly, blood loss is another significant factor. Heavy menstrual periods, blood plasma expansion during pregnancy, and blood loss during childbirth are also listed as potential factors. Furthermore, the role of malaria, fluorosis, and hemoglobinopathies has also been outlined.^9^ Addressing both the nutritional and nonnutritional causes of anemia is integral to the AMB program. However, the scorecard measures only the IFA supplementation received by an individual, which also is the major cause of anemia in the Indian population.

## CONCLUSION

Anemia is a severe public health concern affecting over half of the population across all age groups in India and requires sustained attention and targeted interventions. It remains crucial to enhance IFA supplementation coverage effectively. Regular program reviews are essential to assess performance, prioritize goals, allocate resources, and make informed decisions.

Although the AMB index findings indicate improved IFA supplementation coverage across all groups since the implementation of the AMB program strategy, it is crucial to track the association between coverage and anemia prevalence at the district and subdistrict levels. Regular rankings at these levels can identify gaps, facilitate targeted interventions, and provide a more granular understanding of the program’s implementation and impact.

The observed enhancements in AMB coverage among groups are encouraging, yet sustained advancements are imperative for significantly reducing anemia in India. To achieve this, the AMB strategy must address current challenges and intensify efforts to bolster IFA supplementation coverage in the coming years. Leveraging the AMB index and scorecard as valuable tools for promoting convenience, alongside reporting efficiency, supply status, and implementation status reports, is essential for directing focused initiatives aimed at improving IFA supplementation coverage across groups in India.

The AMB index provides valuable insights into the overall progress and challenges of the AMB program in India and a simple and comprehensive way to evaluate the IFA supplementation coverage across groups. It allows for easy interpretation of progress and enables ranking of states/UTs based on mean coverage. The index can be used to identify well-performing states and areas that need improvement, facilitating targeted interventions and resource allocation to enhance program effectiveness, and can also be used to track progress over time and to evaluate the effectiveness of interventions. By monitoring the AMB index, program managers can identify whether interventions are having the desired impact on IFA coverage.

The AMB index creates an environment of positive competition among states/UTs and districts to achieve and improve the IFA supplementation coverage in all groups. The availability of data on all target individuals is critical for the development of scorecards and accurate monitoring of program progress. States/UTs have embraced the scorecards, and they are using them as a reference tool at national and state/UT levels for anemia-related information.

The successful coverage and distribution of IFA supplements to individuals in the AMB program relies significantly on the coordination and alignment of different departments. This effectively involves synchronization and collaboration between key departments (e.g., Women and Child Development, Health, and Education).

## Notes

**Peer Reviewed**

**First Published Online:** December 10, 2025.
